# The Holistic Effects of Acupuncture Treatment

**DOI:** 10.1155/2014/739708

**Published:** 2014-01-12

**Authors:** Jing-Wen Yang, Qian-Qian Li, Fang Li, Qing-Nan Fu, Xiang-Hong Zeng, Cun-Zhi Liu

**Affiliations:** Acupuncture and Moxibustion Department, Beijing Hospital of Traditional Chinese Medicine Affiliated to Capital Medical University, 23 Meishuguanhou Street, Dongcheng District, Beijing 100010, China

## Abstract

Traditional Chinese Medicine (TCM), as a complex medical science which reflects philosophical principles and embodies large dialectical thought, is used to place the human body into a large system for observation. Acupuncture as a vital part of TCM, has been practiced to treat various diseases and symptoms. However, acupuncture is also facing severe challenges resulted from insufficient modern scientific research. Nowadays, the holistic effects of acupuncture can be researched by some modern approaches, such as the systems biology and fMRI technique. It is believed that having a better understand will greatly promote acupuncture research and be beneficial to scientization and modernization of acupuncture.

## 1. Introduction

Traditional Chinese Medicine (TCM), as a complex medical science which reflects philosophical principles and embodies large dialectical thought, is used to put the human body into a large system for observation [1]. As we knew, there are abundant differences between TCM and modern medicine. For example, health means a state of complete physical, mental, and social well-being in modern medicine, whereas the concept in TCM includes the unison between man and universe, the fusion of shape and soul, the people-oriented view of values, and the balance of *qi-blood-yin-yang* in the human body [2].

Acupuncture, as a vital part of TCM, has been practiced to treat various diseases and symptoms for more than 2500 years and been accepted by the society as a priceless treasure. Despite cultural differences, acupuncture is being used by practitioners in western nations. More and more studies have indicated that acupuncture is safe and effective in treating a wide range of diseases [3–8]. However, acupuncture is also facing severe challenges. One of the reasons is that the methodology used in the acupuncture research is unable to capture the holistic and dynamic nature of diseases [1]. Nowadays, we lacks necessary well-defined molecular mechanism and basis, although acupuncture has been effective in treating many diseases especially chronic illness [2]. To conduct a systemic analysis on human body and diseases under the guidance of holistic view will be an utmost important way for developing acupuncture.

There have been many recent attempts to address these issues but most of them were still based on the “reductionism” philosophy, whereas acupuncture is based on “holism” philosophy. The human body, a complicated system, could be identified as a self-controlled system network. The network is connected by the meridian that exists in whole body. Acupuncture could regulate the balance of human body by the meridians. For example, acupuncture in the Taiyin Lung Meridian of Hand could treat disease of respiratory system. Because the lung is a center together with skin, nose, and large intestine in TCM, acupuncture in the Taiyin Lung Meridian of Hand also could treat skin disease, rhinitis, constipation, and so on. There are some systemic approach appearances to impact our understanding of the theory behind the evidence-based Chinese medicine.

In this paper, we will introduce the holistic effects of acupuncture. A literature review was conducted using PubMed. The keywords consist of (1) “acupuncture,” “acupoint,” or “meridian,” (2) “holistic” or “holism,” (3) “genomics,” “proteomics,” “metabolomics,” “systems biology,” “fMRI,” “brain network,” “PET,” “MEG,” “neuro-endocrine-immune,” “brain-gut axis,” or “hypothalamic-pituitary-adrenal gland axis.” The records retrieved were from the full collections from their inception up to June 2013. A total of 1574 publications were identified as a result of the search. After eliminating 297 duplicated records, 1277 publications remained which were related to the topic.

## 2. Meridian Phenomena with Holistic Theory

According to classic acupuncture theory, there are two opposing and complementary forces that coexist in nature: *Yin* and *Yang*. These two forces interact to regulate the flow of *Qi* (pronounced chee). The traditional concept is usually regarded as energy or life force. When a person is in “good health,” that means *Yin* and *Yang* are in balance and then the flow of *Qi* is smooth. When *Yin* and *Yang* become “unbalanced,” there are disturbances in *Qi*, which lead to illness and disease [9]. The *Qi* circulates through all parts of the body via pathways called meridians, which bring *Qi* from the internal organs to the skin surface. Up to 365 points along and around these meridians which could be stimulated to correct the imbalance and restore the body to normal health are called acupoints [10].

Although the physical evidence for the existence of meridians has not been identified after years of investigation, some meridian phenomena can be found, especially with modern technologies [11]. A number of researchers hold the view that when some internal organs are affected by disease, acupoint sensitization has the potential for exerting dynamic functional changes, reflecting acupoint specificity [12]. Cheng et al. made a model of acute gastric mucosal injury (AGMI) in rats and observed the plasma extravasated Evans blue (EB) points on the skin of the whole body. They found that four acupoints interacting with stomach appeared extravasated EB points. Furthermore, the number of extravasated EB points was related to the phase of gastric mucosal injury, being greatest on the 2nd and 3rd days after modeling and disappearing gradually along with the natural repair of the AGMI [13]. Some research findings suggested that the anatomical structure of meridian channels and acupoints was related to the connective tissues and the connective tissue interstitial fluid (CTIF) system [14–21]. In particular, Yuan et al. analyzed the digital images from slices of cadavers and found that 365 acupoints were located in five types of connective tissues [21]. Dang et al. indicated that 9 out of 11 acupoints of the lung meridian were on the periosteum [16]. Furthermore, another crowd of people devoted to examine the relationship between perivascular space (PVS) and the meridian. According to the perivascular dye injection and frozen section histology, they found that there was PVS around the blood vessels along the meridians, and it is a fluid pathway. Subsequent physiologic studies revealed that the PVS has significantly greater electrical conductivity and significantly higher partial oxygen pressure (pO2) compared to medial and lateral tissues [22].

## 3. Systems Biology in Acupuncture

Systems biology, which combines computational and experimental approaches to analyze complex biological systems, focuses on understanding functional activities from a systems-wide perspective [23]. With the advent of high-throughput genomic, proteomics, and metabolomic technologies, systems biology has become a viable approach for improving our knowledge of health and disease [24, 25]. The suffix “-omics” is added to the object of study or the level of biological process to form new terms to describe that information. For example, genomics from gene data, proteomics from protein data, and metabolomics from metabolic data [26]. Omics data helps to explore the different levels in systems biology from a holistic perspective. The area of integrating acupuncture with systems biology approach has become a major hot of TCM research. Recent advances in systems biology technology have enabled the discovery of biomarkers, potentially offered “the right therapy for the right patient” [27].

### 3.1. Genomic Studies of Acupuncture

Genomics is an approach collecting information from genomes to guide medical decision making and to tailor strategies for each patient. Nowadays, many individual transcriptional profiles of animals or patients have been mined to search for target molecules of acupuncture treatments. Meanwhile, candidate genes or pathways associated with the protective effect of acupuncture treatments have been revealed through genomic analysis for several diseases and symptoms [28]. The microarrays of either cDNA or oligonucleotide probes were used to screen for potential candidate genes to mediate acupuncture responses [29]. Lots of studies proved that acupuncture had many holistic effects by regulating relative genes (Table 1).

### 3.2. Proteomic Studies of Acupuncture

Proteomics is the information of a whole proteome, which refers to the entire complement of proteins within an organism or system [30]. Proteomics technology is based on the vast analytical power for protein, peptide identification, and quantification offered by two-dimensional electrophoresis (2-DE) and various mass spectrometry (MS) techniques. In a growing number of study, researchers have reached a number of achievements on investigating the mechanisms of acupuncture by using differential proteomics (Table 2).

### 3.3. Metabolomics Studies of Acupuncture

Metabonomics is a newly emerging modern technology in the postgenome era and has been being used widely in the study on TCM [31]. Nuclear magnetic resonance (NMR) is one of the key techniques used to acquire massive dynamic and quantitative information about small molecular weight metabolites in the body. As a systemic approach, metabolomics is able to employ a “top-down” strategy to measure the function of organisms from the end products of the metabolic network and to explore the metabolic changes triggered by interventions at the whole-system level in a holistic context [1]. Nowadays, more and more researchers focus on this field (Table 3).

As we can see, the practice of holistic acupuncture shares similarities with many basic concepts of systems biology. Acupuncture treats diseases not by modulating the location of illness but by regulating the gene, protein, or metabolite, which can influence the whole body at the organismal level.

## 4. The Brain Networks Influenced by Acupuncture

The brain has two hemispheres, each of which has four lobes: the frontal, temporal, parietal, and occipital lobes. The frontal lobes are regarded as the executive center and are involved in working memory, planning, and cognitive evaluation. The temporal lobes are implicated in evaluative processing and memory. The parietal lobes are most often involved in spatial processing, whereas the occipital lobe mainly supports vision. Moreover, the primary somatosensory cortex (SI) is the most important area for sensing touch in the cortical brain [56]. Acupuncture as a treatment is widely used in the world; its physiological mechanism is not clear and needs further investigation. In recent years, there has been a growing number of evidence demonstrating the response of the central nervous system (CNS) to acupuncture, and several functional imaging studies have revealed the specific activities of CNS during acupuncture [57]. To discuss the neurobiological mechanisms of acupuncture, a large proportion of neuroimaging researches have been carried out with the utilization of functional magnetic resonance imaging (fMRI), positron emission tomography (PET), and magnetoencephalography (MEG). Through our review, fMRI is the most common measure to be used (309 out of 362 papers), while 46 papers employed PET and 7 papers adopted MEG. It is well known that fMRI is the most common technique among neuroimaging. The wide range of physical effects exerted by acupuncture suggest that the brain may be responsible for transmitting the needle stimulus into signals aiming at maintaining homeostatic balance within and across functional subsystems. No matter either of the three techniques, it has opened a “window” into the brain, allowing us to investigate the central physiological functions involved in acupuncture administration of human beings available [12, 13, 58].

### 4.1. Different Diseases in Acupuncture

Maeda et al study the linkage between brain response to acupuncture in chronic pain patients with carpal tunnel syndrome (CTS). They found that electroacupuncture (EA) applied at local acupoints on the affected wrist PC7 (Daling) to SJ5 (Waiguan) produced greater activation in insula and secondary somatosensory cortex (SII) and greater deactivation in ipsilateral SI, while distal EA applied on the contralateral ankle SP6 (Sanyinjiao) to LR4 (Zhongfeng) produced greater activation in SII and deactivation in posterior cingulate cortex. These regions mentioned above were correlated with pain reduction following stimulation [59]. Napadow et al. showed that during an increasing itch phase, activation was localized in anterior insula and striatum, regions associated with salience/interoception, and motivation processing. Greater itch reduction following acupuncture was associated with greater reduction in putamen response, a region implicated in motivation [60]. Feng et al. investigated the effect of acupuncture in Alzheimer's disease (AD) and mild cognitive impairment (MCI) patients by combing fMRI and traditional acupuncture. They found that after acupuncture, there are several regions showing increased or decreased activities in MCI, AD subjects compared to normal subjects. Most of the regions were involved in the temporal lobe and the frontal lobe, which were closely related to the memory and cognition. Their fMRI study confirmed that acupuncture at LR3 (Taichong) and LI4 (Hegu) could activate certain cognitive-related regions in AD and MCI patients, such as the left SFG, the left MFG, bilateral IFG, left MTG, the left lentiform nucleus, the left temporal lobe, and left MFG [61].

### 4.2. Different Puncture and Stimulation Methods in Acupuncture

Napadow et al. compared the central effects of EA at different frequencies with traditional Chinese manual acupuncture. In their experiment, manual acupuncture, EA at 2 Hz and 100 Hz, and tactile control stimulation were carried out at acupoint ST36 (Zusanli). Overall, EA (particularly at low frequency) produced more widespread fMRI signal increase than manual acupuncture did, such as anterior middle cingulate cortex, pontine raphe area [62]. Quah-Smith et al. examine the difference of laser and needle acupuncture in relation to brain effects of activation of LR8 (Ququan), a putative acupuncture point for depression. They found that laser acupuncture activated the precuneus relevant to mood, while needle acupuncture activated the parietal cortical region associated with the primary motor cortex [63]. Fang et al. used fMRI in 15 healthy subjects to investigate cortical activation during rotating or nonrotating stimulation method. Compared to the non-rotating stimulation method, they observed an activation in SII, frontal areas, the right side of the thalamus, and the left side of the cerebellum during rotating stimulation [64]. Wang et al. applied fMRI to investigate the neural correlates of individual components of Deqi during acupuncture on the right LR3 acupoint. Bilateral limbic-paralimbic-neocortical network (LPNN), right orbitofrontal cortex, and bilateral posterior parietal cortex were found to be responding to Deqi [65].

### 4.3. Different Durations in Acupuncture

Li et al. aimed at evaluating the effects of manual acupuncture with different durations on the human brain using fMRI. The results showed that longer stimulation (180 sec) could induce broader CNS responses than shorter acupuncture (30 sec; 60 sec), for example; the activations in occipital lobe, cerebellum, the deactivations in dorsal lateral prefrontal cortex (DLPFC), and so on [57].

Several conclusions can be made based on the above fMRI studies. First, the SII may play a vital role in acupuncture analgesia. Second, traditional acupuncture and EA at the same acupoint activate different regions, and EA produced more widespread fMRI signal increase than manual acupuncture did. Third, acupuncture at any points, not just in the brain, could stimulate different regions and then modulate various diseases.

Future studies that evaluate both central and peripheral effects of needle stimulation, in a well-chosen disease model, may help determine specifically which acupuncture effects are most important to clinical efficacy.

## 5. Neuroendocrine-Immune Functional System in Acupuncture

Three regulating systems, nervous, endocrine, and immune, are involved in maintenance of homeostasis. They are regarded as interacting, with mutual effect between nervous and endocrine systems being well defined and giving rise to development of independent realm of knowledge—the neuroendocrinology. Meanwhile, the interaction between the neuroendocrine and immune systems seems to be exciting and intensely developing trend of up-to-date investigation. There are some evidence adduced bringing new sight into the mechanisms of bidirectional exchange of signals among the nervous, endocrine, and immune systems [66].

### 5.1. The Brain-Gut Axis

The brain and the gastrointestinal system communicate through a two-way connection known as the “brain-gut axis.” In this axis, the CNS releases neurotransmitters to guide the esophagus, stomach, and intestines when to produce secretions and/or when to move. The gastrointestinal tract in turn sends chemical and electrical signals in response to the brain, which can be regarded as sensations of hunger, pain, and so on [67–69]. Increasing knowledge acquired from animal models detecting the brain-gut axis has provided potential insight into the management of inflammatory bowel disease in humans [70]. Eshkevari et al. found that EA attenuates visceral hyperalgesia in a central rat model of irritable bowel syndrome. The antihyperalgesic effect is probably mediated by downregulation of serotonergic activities in CNS [71].

### 5.2. The Hypothalamicpituitary-Adrenal Gland (HPA) Axis

The HPA axis consists of the hypothalamus, the pituitary, and the adrenal glands in which corticotropin-releasing hormone (CRH), adrenocorticotropic hormone (ACTH), and cortisol secreted; respectively, interact through receptor dynamics [72]. Park et al. investigated the ability of acupuncture at acupoint HT7 (Shenmen) on early life stress. In this study, acupuncture reduced anxiety-related behaviors in maternal separation (MS) rats, and decreased corticosterone and ACTH levels in plasma of MS rats. They demonstrated that acupuncture at HT7 protected MS-induced anxiety-related behaviors and activated the HPA system [73].

### 5.3. The Hypothalamopituitary-Gonadal (HPG) Axis

The HPG axis comprises the hypothalamic gonadotrophin-releasing hormone- (GnRH-) anterior pituitary luteinising hormone (LH) and follicle stimulating hormone- (FSH-) ovarian oestrogen cascade [74]. Zhaohui et al. found that the repeated low frequency EA (3 Hz) downregulated HPG axis of common rats and rabbits during puberty. In their latest study, they investigated the role of neuropeptide Y (NPY), an important regulator of HPG axis, in EA-treated rats. The results showed that repeated low frequency EA was an effective method on down-regulating not only the GnRH expression but also the NPY expression in the hypothalamus during early puberty of rats [75].

There is an increasing number of new molecular and neurophysiological research reports in various aspects, such as anti-inflammatory immune response and neuroimmune response. A recent series of studies conducted by Tracey and colleagues described the interaction between the autonomic nervous system (ANS) and the immune functions. Inflammatory information is transmitted through sensory nerves to the hypothalamus where input signals are processed; it then results in an anti-inflammatory output via the ANS. They thought that acupuncture might be involved as a modulator of the immune system [76, 77]. Although actual scientific evidence is yet to be scrutinized, studies concerning neuroimmunology and autonomic reflexes could form an important base for understanding of the basic acupuncture mechanism as a neural-immune reflex [78, 79].

## 6. Conclusion

Western medicine differs many aspects from TCM, including in its guidelines, practical bases, and approaches in treating diseases [80]. One hand, western medicine is based on human anatomy, biology, biochemistry, and molecular biology. It mainly relies on the analysis of lab results for diagnosis and treatment of the visible human body and the solid evidence of an illness. In contrast, TCM, which theorizes that “any internal disease will, in one way or another, reflect on the *Zheng* (symptom), the outer look of the human body” [81], focuses more on direct contact with patients. TCM can be characterized as holistic with emphasis on the integrity of the human body and the close relationship between human and its social and natural environment. On the other hand, western medicine belongs to allopathic medicine, a facet of experimental science in typical western culture. A disease caused by cell and tissue infection is often treated by chemical or physical repair, removal, insertion, bypass, stents, or transplant of organs or tissues. TCM that focuses on the meridian and other energy flow systems holds that all diseases originate from the imbalance of qi and blood flow [82]. Hence, this holistic emphasis on body functions and the spirit is a comprehensive approach.

In this review, we described and discussed lots of effects produced by acupuncture, performed at the holistic view level. In summary, acupuncture offers multiple holistic approaches and potentially impacting on major human diseases, and it regulates the balance of body in molecular level. These approaches will facilitate the practice of acupuncture through a variety of methods. Then acupuncture can be developed even further and provide important information for therapeutic strategies in managing various diseases and conditions.

In TCM, the holistic view not only means the harmonious unity of the whole body, but also includes the unison between man and environment. Therefore, future acupuncture studies are needed to investigate various input parameters which can affect the outcome of acupuncture stimulation, such as the timing of treatment, the temperature of treating room, the intensity, frequency and duration of stimulation, and the repetition rate. In addition, various physiological differences, such as body constitution, daily rhythm of humoral secretion (such as glucocorticoids), and pathological conditions should also be considered as important parameters to which attention must be paid.

Enormous challenges remain at the present time, but one can foresee that the application of technologies mentioned above in the clinical practice will eventually lead to the reconciliation and integration between acupuncture and contemporary medicine.

## Figures and Tables

**Table 1:** Summary of experimental studies of acupuncture articles on genomic technologies.

Author/year	Treatment manner	Disease	Acupoint	Testing technique	Upregulation gene	Downregulation gene
Li et al./2012 [32]	Electroacupuncture	Hypercholesterolemia	ST40 (Fenglong)	cDNA microarray	NM_013474, NM_008138, NM_008712, and so forth (18 genes)	NM_133668, NM_007409, NM_011407, and so forth (13 genes)

Choi et al./2011 [33]	Acupuncture	Parkinson's disease	GB34 (Yanglingquan); LR3 (Taichong)	cDNA microarray	Atp2a1, Dub2a, Klhl31, and Tnnt3.	Hba-a1, Hba-a2, Hbb-b1, EG383229, Ppbp, and Ube2l6

Shiue et al./2010 [34]	Acupuncture	Allergic rhinitis	LI4 (Hegu); ST36 (Zusanli); LI20 (Yingxiang); EX-HN3 (Yintang)	Microarray	the most upregulated genes in Ph (−) patient	The most downregulated genes in Ph (+) patients

Tan et al./2010 [35]	Warm needling	Knee osteoarthritis	RN4 (Guanyuan); RN6 (Qihai); ST36; EX32 (Xiyan); GB34	cDNA microarray	14, 45, 11, and 31 genes	16, 56, 20, and 10 genes

Sohn et al./2010 [36]	Electroacupuncture	Immunomodulation	ST36	Microarray	Sod1, IL1f9, Oprl1, and Oprk1	Cyp2a4, ITGA4, and Foxf2

Wang et al./ 2009 [37]	Electroacupuncture	Spinal cord injury (SCI)	ST36; GB39 (Xuanzhong); ST32 (Futu); SP6 (Sanyinjiao)	Microarray	CNDF, FGF-13, FGF-1, IGF-1R, CGRP-*α*, and NPY	CNDF (1dpo), CNDF (14dpo), FGF4, IGF1 (14dpo), TGF*β*2, and PF75

Shiue et al./ 2008 [38]	Acupuncture	Allergic rhinitis	LI4; ST36; LI20; EX-HN3	cDNA microarray	9 genes	72 genes

Gao et al./2007 [29]	Electroacupuncture	Pain	ST36	Oligonucleotide microarray	NM_021669, NM_013099, NM_057207, NM_053868, NM_131912, AF255612, NM_078620	N.A.

Li and Zhang/ 2007 [39]	Electroacupuncture	Hypercholesterolemia	ST40	Oligo microarray	Gdf15, Ppap, and OATP-1	Slpi, LXR

Ding et al./2006 [40]	Acupuncture	Aging	CV17 (Shanzhong); CV12 (Zhongwan); CV6 (Qihai); ST36; SP10 (Xuehai)	cDNA microarray	Hsp84, Hsp86, and YB-1	N.A.

Kim et al./2005 [41]	Electroacupuncture	Natural killer cell activities	ST36	Oligo microarray	PTK, VCAM-1	PTP, SHP-1

Guo et al./2004 [42]	Electroacupuncture	Ischemia	GV26 (Renzhong); GV20 (Baihui)	cDNA microarray	27 genes	2 genes

Ko et al./2002 [43]	Electroacupuncture	Neuropathic pain	ST36	cDNA microarray	Opioid receptor, MAP kinase, zinc finger protein, and tyrosine phosphatase related genes	N.A.

**Table 2:** Summary of experimental studies of acupuncture articles on proteomic technologies.

Author/year	Treatment manner	Disease	Acupoint	Testing technique	Upregulation protein	Downregulation protein
Bae et al./2013 [44]	Acupuncture	Kainic acid-induced neuronal destruction	HT8 (Shaofu)	2-dimensional electrophoresis	VCP	ULMAE-1, ATPS, HSP70, HSP4L, and CRMP-2

Lai et al./2012 [45]	Acupuncture	Hypertension	LR3	2-dimensional electrophoresis and MALDI-TOF	Glutamate dehydrogenase 1, aldehyde dehydrogenase 2, glutathione S-transferase M5, Rho GDP dissociation inhibitor 1, DJ-1 protein, and superoxide dismutase	Heat shock protein-90, synapsin-1, pyruvate kinase isozyme, NAD-dependent deacetylase sirtuin-2, protein kinase C inhibitor protein 1, ubiquitin hydrolase isozyme L1, and myelin basic protein

Gao et al./2012 [46]	Electroacupuncture	Chronic constrictive injury	ST36; GB34	2-dimensional electrophoresis and matrix-assisted laser desorption	19 hippocampal proteins which are involved in metabolic, physiological, and cellular processes	

Pan et al./2011 [47]	Electroacupuncture	Acute ischemic stroke	MS6 (scalp acu motor areas); BL10 (Tianzhu); GB20 (Fengchi); LI4; PC6 (Neiguan); BL40 (Weizhong); SP6 (Sanyinjiao); ST36	2-DE + MS/MS	Gelsolin, C3	Serpin G1

Kim et al./2010 [48]	Acupuncture	Maternal separation	HT8	2-dimensional electrophoresis and MALDI-TOF	Dpysl2, Drp2, Tuba1a, and Stx1b	N.A.

Li et al./2010 [49]	Electroacupuncture	Spinal cord injury (SCI)	GV6 (Jizhong); GV9 (Zhiyang)	2-dimensional electrophoresis and MALDI-TOF	ANXA5, CRMP2	N.A.

Kim et al./2010 [50]	Electroacupuncture	Parkinson's disease	GB34; GB39 (Xuanzhong)	2-dimensional electrophoresis and MALDI-TOF	Cytochrome coxidase, subunit Vb	HAGH, cytosolic malate dehydrogenase Munc 18-1, hydroxyacyl glutathione, and hydrolase

Jeon et al./2008 [51]	Electroacupuncture	Parkinson's disease	GB34; SI3; BL62; ST36	2-dimensional electrophoresis	21 proteins	1 protein

**Table 3:** Summary of experimental studies of acupuncture articles on Metabolomics technologies.

Author/year	Treatment manner	Disease	Acupoint	Testing technique	Upregulation metabolite	Downregulation metabolite
Wu et al./2011 [52]	Electroacupuncture	Aging	GV 20 (Baihui); KI1 (Yongquan)	NMR	Lactate, DMA, choline, and TMAO	N.A.

Wu et al./2010 [53]	Electroacupuncture	Functional dyspepsia (FD)	BL21 (Weishu); CV12	NMR	VLDL/LDL	NAc

Tang et al./2009 [54]	Electroacupuncture	Aging	GV20; KI1	NMR	saturated fatty acid; triglyceride	Choline; phosphatidylcholine; unsaturated fatty acid

Wu et al./2008 [55]	Electroacupuncture	Chronic emotional stress	GV20; SP6	NMR	Nsaturated fatty acid; phosphatidylcholin	Glucose; VLDL
